# Healing Influence of *Melilotus Officinalis* Herbal Extract on Experimental Autoimmune Encephalomyelitis in C57BL/6 Mice

**DOI:** 10.22037/ijpr.2020.113808.14505

**Published:** 2020

**Authors:** Mahdieh Hassani, Maryam Soleimani, Emran Esmaeilzadeh, Davood Zare-Abdollahi, Hamid Reza Khorram Khorshid

**Affiliations:** a *Genetics Research Center, University of Social Welfare and Rehabilitation Sciences, Tehran, Iran. *; b *Department of Basic Sciences, University of Social Welfare and Rehabilitation Sciences, Tehran, Iran. *; c *School of Medicine, AJA University of Medical Science, Tehran, Iran.*

**Keywords:** Multiple sclerosis, Cytokines, EAE, Melilotus Officinalis

## Abstract

The present study was designed to primarily examine the therapeutic potential of the herbal extract of *Melilotus Officinalis* for the treatment of multiple sclerosis in the experimental autoimmune encephalomyelitis (EAE) model of the disease. The animal model was induced in C57BL/6 female mice, and then the herbal extract was intraperitoneally administered for a total of 21 days after the first day of post-immunization. The phenotypic signs, a gene expression profile of inflammatory cytokines, antioxidant state, and pathological hallmarks of the disease in the corpus callosum were evaluated. The prophylactic administration of *Melilotus Officinalis* attenuates the clinical signs of the disease. It significantly declined the gene expression of pro-inflammatory cytokines like IL-6, TNF-α, and IFN-γ. This herbal extract also surged the gene expression, as an anti-inflammatory cytokine. The gene expression of Glutathione peroxidase and Catalase (antioxidant enzymes) was meaningfully higher in the treatment group. Pathological evaluation of corpus callosum cross-sections by Luxol Fast Blue staining revealed preserved myelin sheath in the treated group compared to the EAE mice. The results of our assay confirmed that immunomodulatory and antioxidant features of the herbal extract of *Melilotus Officinalis* ameliorated the EAE severity. This study finding disclosed the therapeutic efficiency of this compound in MS treatment.

## Introduction

Multiple sclerosis (MS) is an insidious neurodegenerative disease that affects the brain and spinal cord. MS undoubtedly represents the most frequent cause of neurological disability that begins in early to mid-adulthood (1). This disease is popularly considered as an autoimmune disorder that inflammation and neurodegeneration are underlying causes of its development. Inflammatory focal lesions, primary demyelination, and reactive gliosis in the CNS remain pathological features of MS (2). Although the exact etiology and pathogenesis of MS are not clear yet, influential factors, such as genetic and environmental factors, immune system dysfunction, and a stressful lifestyle are involved (3, 4). The disease can start as episodic attacks or sustained progress. Oxidative damage to oligodendrocytes, oligodendrocyte apoptosis, and disruption of Th2/Th1 cytokine balance play critical roles in the pathogenesis of MS. While pro-inflammatory cytokines, such as IL-6, TNF-α, IL-12, and IFN-γ are secreted by Th1 cells, Th2 cells release anti-inflammatory cytokines like IL-4, IL-5, and cytokines are critical elements in the progression of inflammation and lead to axonal damage and brain lesions (5, 6). 

Although there is no permanent cure for multiple sclerosis, existing therapies are only disease-modifying (DMT). Typically, the primary goals of treatment are rapid recovery after an attack, prevention of new attacks, and avoidance of disability (7). Recent research indicates the beneficial effects of herbal extracts in the treatment and prevention of MS disease (8, 9). *Melilotus Officinalis* is an herbal medicine with antioxidant and anti-inflammatory properties that grows abundantly in Iran (10). *Melilotus Officinalis* extract contains two notable groups of phenolic compounds, including 7-hydroxy coumarin and flavonoids that were initially considered effective in treating diabetic foot ulcers (11, 12). According to biochemical and pathological studies, no toxic effects have been observed in this drug (13-16). This medicine remarkably improves oxidative stress and reduces inflammatory cytokines TNF-α, IL-6, NF-kB, and IL-1β in the aging mouse model (17). Past studies have also shown that *Melilotus officinalis* extract sufficiently reduces nitric oxide synthesis (10). 

According to those above, this study aimed to evaluate the antioxidant and immunomodulatory effects of *Melilotus Officinalis* herbal extract in the C57BL/6 mouse model of experimental autoimmune encephalomyelitis (EAE). The target tissue in this study was corpus callosum, which is the most critical connector between the two cerebral hemispheres.

## Experimental

*Animal, Model induction, and Treatment*

Thirty-two adult female C57BL/6 mice (10-12 weeks, 18-20 g.) were obtained from Pasteur Institute of Iran and randomly divided into four experimental groups including: 1) control (healthy mice), 2) EAE, 3) sham (EAE + vehicle), and 4) treatment group. Each group was kept in a separate and standard cage. The animals were allowed to adapt in an isolated room under constant temperature (25 °C), and intermittent 12 h dark/light for a week. Animal Research Ethics Committee of the University of Social Welfare and Rehabilitation Sciences approved all procedures of our experiment (IR.USWR.REC.1397.161). 

Briefly, EAE was induced in C57BL/6 mice with Hooke kit (Hooke Laboratories, Inc, USA) by the subcutaneous injection of 300 µg of emulsified MOG35-55 peptide in 0.1 ml PBS plus an equal amount of Incomplete Freund’s Adjuvant containing 4 mg/mL of Mycobacterium tuberculosis. Then, 500 ng of Bordetella pertussis toxin was injected intraperitoneally on the first day of the model induction and on the second day (48 h after the first injection). No treatment was performed in the control and EAE groups. In the treatment group, 10 mg/kg of the herbal extract compound (that obtained by Nano Hayat Darou company, Iran) was daily injected via IP from day one post-immunization for 21 days. The sham group just received the vehicle (dilution of 82% ethanol) of the drug.

*Assessments*

*Scoring*

Typically, the gradual onset of signs of EAE begins between days 10 and 12 post-immunization. After the disease signs appear, the animals were daily scored based on the following criteria: no symptom (score = 0); partial tail paralysis (score < 1); tail paralysis and partial hind limb numbness (1 ≤ score < 2); tail paralysis, hind limb numbness and apparent waddling gait (2 ≤ score < 3); complete hind limb paralysis (3 ≤ score < 4); tail paralysis, complete hind limb paralysis and forelimb numbness (3 ≤ score < 4); tail paralysis and quadriplegia (4 ≤ score < 5), death (score = 5). 

Moreover, animal weight, as an objective indicator of disease progression, was measured daily.

*Grip Strength*

Test is commonly a non-invasive *in-vivo* method to properly evaluate the weakness of muscles. Based on this method, each mouse was allowed to tightly hold the triangular bar of grip strength meter (Columbus Instruments), then pulled away horizontally at a constant speed. The test was repeated three times for each mouse, and the result of the best performance was recorded.

*Gene Expression Evaluation*

 Total RNA was carefully extracted from the corpus callosum tissue using the Hybrid-R^TM ^RNA isolation kit (GeneAll, South Korea), and then the quality and quantity of extracted RNA were determined by agarose gel electrophoresis and NanoDrop ND-2000 spectrophotometer (Thermo Fisher Scientific, Wilmington, USA), respectively. 500 ng of extracted RNA was reverse-transcribed to cDNA via the PrimeScript™ RT reagent Kit (Takara, Japan). Specific primers for target genes were uniquely designed using the NCBI database and primer3 software. The necessary information of primers was listed in (Table 1).

Finally, real-time PCR reaction for target and internal control genes was performed using the SYBR Green Master Mix protocol and kit (Ampliqon, Denmark). In this experiment, duplicate qPCR reactions were efficiently performed in 40 cycles using the ABI 7500 instrument (Applied Biosystems, Foster City, CA, USA). 

The relative expression value of target genes was normalized with HPRT-1 as an internal control and calculated using the 2^-ΔΔCt^ method, and fold change expression between groups was computed. 

*Histopathological Process*

For histopathological examination, two mice from each group were used. The animals were anesthetized by intraperitoneal injection of ketamine (20 mg/kg) and xylazine (0.64 mg/kg). The animals were perfused and fixed with 0.1% PBS and paraformaldehyde (PFA) and kept in 4% PFA for 48 h (post-fixation) and then processed and blocked. Next, the paraffin blocks were coronally cross-sectioned 5 µm by rotary microtome (Leica microsystem RM2235, UK), and 20 sections of corpus callosum tissue were obtained. To evaluate demyelination, the samples were stained with Luxol Fast Blue (based on Kluver-Barrera method), and after staining, the samples were analyzed by light microscope and Image J software.

*Glutathione peroxidase Enzyme Activity Assay*

Glutathione Peroxidase (GPX-1) catalyzes the glutathione (GSH) oxidation reaction by Cumene Hydroperoxide. In the presence of glutathione reductase enzyme and NADPH, oxidized glutathione (GSSG) is reconstituted into reduced glutathione (GSH), which is accompanied by the simultaneous oxidation of NADPH to NADP +. In this reaction, the light absorption is measured at 340 nm. The measurement of NADPH oxidation reflected the enzyme activity level of GPx. Briefly, a solution mixture of glutathione, sodium phosphate buffer (pH 7.2), EDTA, and glutathione reductase was prepared in this assay. Then the samples were incubated in the solution for 4 min. Afterward, NADPH was added, and the absorbance was measured at 340 nm over 3 min.

*Statistical Analysis*

The data of histopathological and enzymatic activity assessments were analyzed by GraphPad Prism software version 8 (La Jolla, Ca, USA. Student *t-*test was used for the comparison of two groups and ANOVA was used for comparison of more than two groups. The efficiency values of qPCR reactions were determined by the LinReg method. REST 2009 software was used to calculate gene expression levels. The *P*-value of less than 0.05 was statistically significant.

## Results

*Comparison of Phenotypic Assessments between Different Groups*

**Figure 1:**
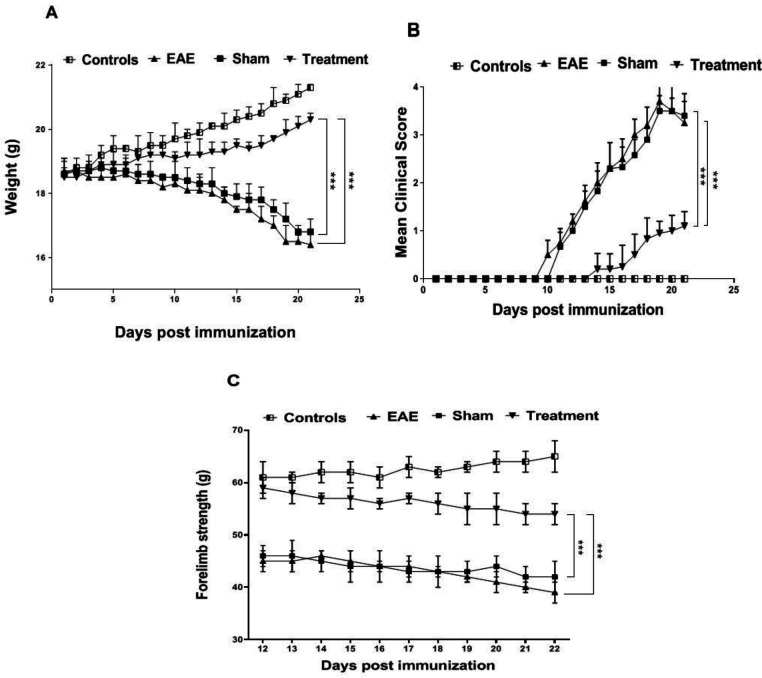
Effect of prophylactic administration of *Melilotus Officinalis* extract on EAE severity and onset (A) The average weight of the mice through evaluation (B) Mean clinical score of groups (C) Grip-strength test data**. **The results are the means ± SEM of data (***P < 0.001)

As losing weight is one of the relevant indicators of disease progression in EAE, the mice were weighed daily following the induction(18). Our investigation showed a significant decrease in the weight of EAE and sham groups compared to the controls (*P* < 0.001). Results of the treatment group demonstrated a significant effect of *Melilotus Officinalis* on losing weight in comparison to the EAE and sham groups (Figure 1A,* P *< 0.001).

The scoring results are considered as one of the most critical features to confirm EAE modeling. In the EAE and Sham groups, the maximum average score was 3.7 and 3.5, respectively. This value was 1.1 for the treated animals. Whereas the onset of the symptoms in the EAE group was on day 11 after immunization, the symptoms appeared on day 14 in the treatment group. These results also indicated a remarkable effect of *Melilotus Officinalis* on the attenuating of disease progression (Figure 1B,* P *< 0.001).

To evaluate the forelimb strength properly, the animals were monitored daily from the 10 th day after immunization, and the results were recorded. Generally, due to the progression of the disease in animals, their muscle strength is impaired. In this study, on day 21 after model induction, forelimb power in the EAE and sham groups significantly decreased compared to the control ones (*P* < 0.001). Appraisal, the treatment group showed a notable increase in muscle strength compared to the EAE and sham groups (Figure 1C,* P *< 0.001). 

*The Results of Cytokines Gene Expression Evaluation*

**Figure 2:**
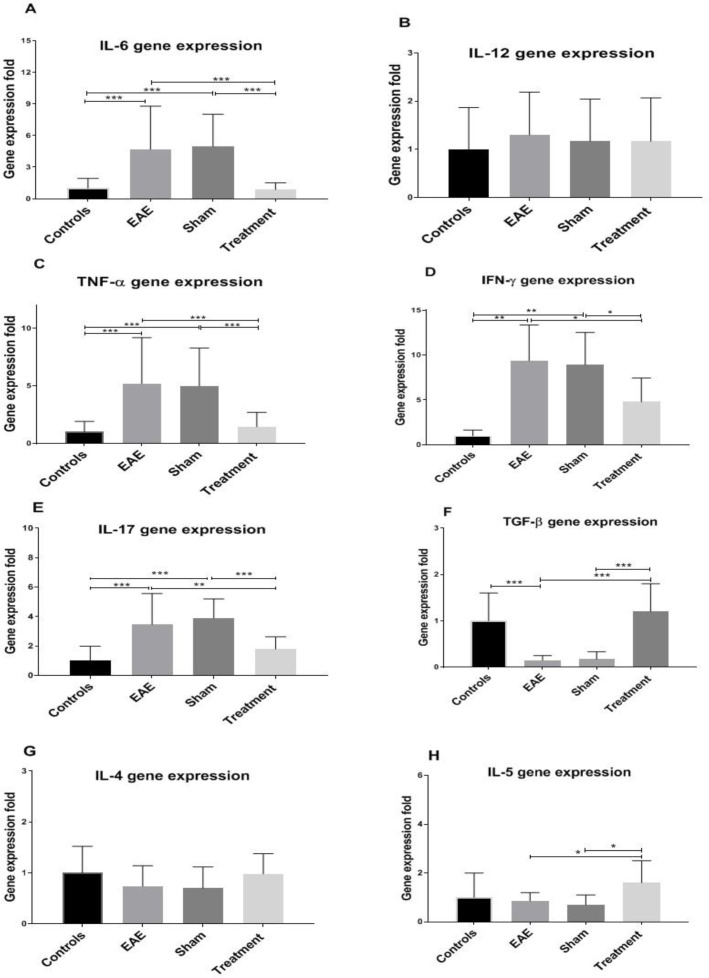
The effects of *Melilotus Officinalis* treatment on the cytokines coding gene expression. (*P < 0.05, **P < 0.01, ***P < 0.001). The results are the means ± SEM of data

The expression level of pro-and anti-inflammatory genes was evaluated in the corpus callosum samples of various groups. Statistical analysis revealed a significant increase in the expression level of the pro-inflammatory cytokines (IL-6, TNF-α, IFN-γ, and IL-17) in the EAE and sham groups compared to the controls (Figure 2, all* P *< 0.001). On the other hand, the expression of the anti-inflammatory cytokine, in the non-treated groups indicated a decrease compared to the control mice (Figure 2F,* P *< 0.001). Interestingly, *Melilotus Officinalis* treatment significantly reduced the expression of pro-inflammatory cytokines, except IL-12 (Figure 2). Besides, *Melilotus Officinalis *treatment elevated the expression of TGF-β and IL-5 (twofold), anti-inflammatory cytokines, compared to the EAE mice (*P* < 0.001 and* P *< 0.05, respectively). The expression of IL-4 was not statistically significant (Figure 2G).

*Investigation of Antioxidant Capacity Changes*

**Figure 3:**
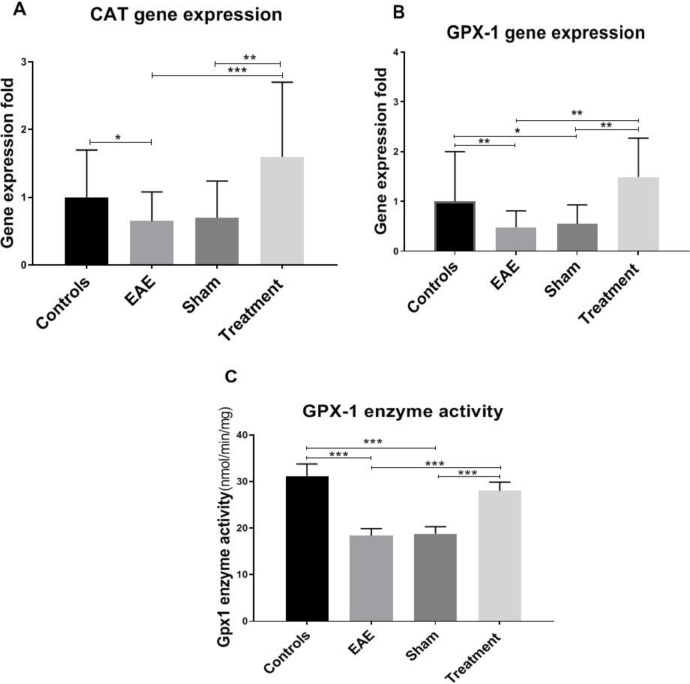
Effects of *Melilotus Officinalis* treatment on antioxidant status. (A) *CAT* genes expression, (B)* GPX-1 *genes expression (C) antioxidant enzyme activity (*P < 0.05, **P < 0.01, ***P < 0.001). The results are the means ± SEM of data

To examine the effect of *Melilotus Officinalis* treatment on provoking antioxidant status, Catalase (CAT) and Glutathione peroxidase (GPX-1) gene expression levels were evaluated. The results of this study showed a significant decline in the expression level of CAT and GPX-1 genes in the non-treated groups compared to the controls (*P* < 0.05 and* P *< 0.01, respectively). Treatment with *Melilotus Officinalis* remarkably increased the gene expression of these two enzymes (Figure 3, *P *< 0.01 and *P *< 0.001, respectively). Besides, the GPX-1 enzyme activity was assessed. Our data indicated a notable decrease in the GPX-1 enzymatic activity due to EAE model induction, which is surged up with *Melilotus Officinalis* administration. (Figure 3C). 

*Histopathological analysis of Corpus Callosum*

**Figure 4:**
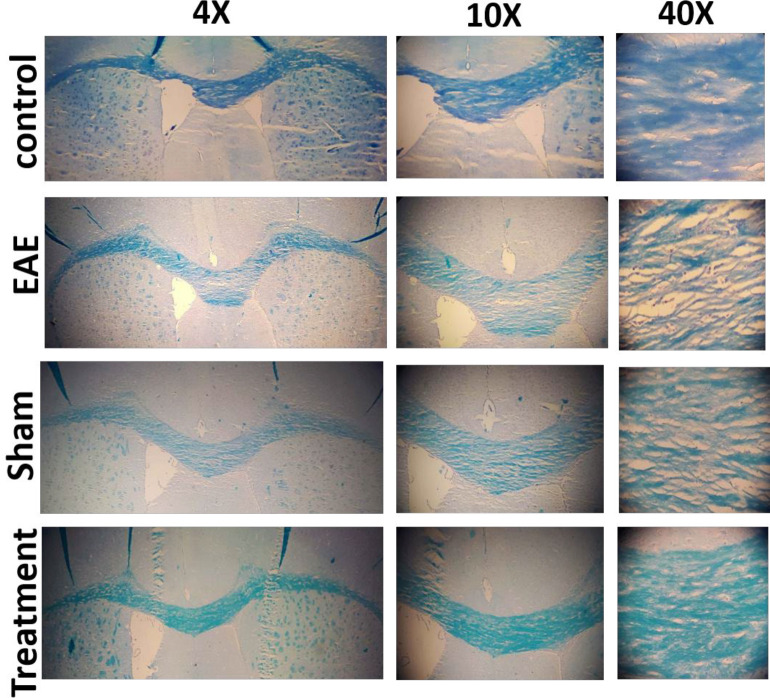
Effects of *Melilotus Officinalis* treatment on the demyelination area in Corpus callosum at different magnifications. Demyelination areas are shown with arrow

The beneficial effect of *Melilotus Officinalis* treatment on demyelination and myelin retention in the corpus callosum was investigated. The expansion of demyelination was assessed using Luxol Fast Blue staining, and the primary images were accurately quantified by ImageJ software. The results showed a significant enhancement of demyelination in the EAE and sham groups (both* P *< 0.001). Prophylactic administration of the herbal compound significantly restricted the demyelination rate and preserved myelin in the corpus callosum (Figure 4, *P *< 0.001).

## Discussion

According to several studies, the leading role of cytokines in inflammatory and autoimmune diseases is undeniable. The type of cytokines and their complex function undoubtedly have a critical influence on the process and the outcome of these types of disorders (19, 20). A scientific consensus about the autoimmune origin of MS is that the imbalance between inflammatory and anti-inflammatory cytokines determines the progression of inflammation and the severity of the disease (21, 22). Since most of the evidence proposes the role of inflammatory cytokines as one of the primary causes of MS, the therapeutic approach has tended to anti-inflammatory drugs (19). On the other hand, studies have corroborated the function of oxidative stress on the pathophysiology of MS disease. The decrease in the antioxidant activity in the blood of MS patients has been indicated (23). Cytokines as major mediators of inflammation and crucial inducers of oxidative stress play a critical role in MS. Therefore, in this primary study, the beneficial effect of the herbal extract of *Melilotus Officinalis* on ameliorating acute inflammation and reducing oxidative stress in the EAE model of MS was investigated. The *Melilotus Officinalis* influence on the betterment of phenotypic and histopathological symptoms of EAE was also checked. Our assay convincingly showed that this herbal compound improved phenotypic symptoms and prevented losing weight in the treatment group compared to the EAE mice. Besides, the results of the forelimb strength test showed that the treated EAE mice had adequate muscle strength and to some extent were similar to the control animals.

Prior studies have confirmed that the accumulation of cytokines, such as TNF-α, IFN-γ, and IL-6 is important mediators of MS pathology. Moreover, their accumulation is related to demyelination and damage to oligodendrocytes and ultimately lead to neuronal death. Therefore, in this study, the effect of *Melilotus Officinalis* treatment on the demyelination, as a critical disease index, was investigated. Histopathological results of corpus callosum tissues clarified that *Melilotus Officinalis* was able to prevent demyelination. The data in )Figure 4( described the relative preservation of myelin in the treatment group compared to the EAE and Sham groups. Anti-apoptotic and neuroprotective effects of this herbal extract were demonstrated in the brain of ischemic rats (24). Moreover, previous evidence of its protective properties on myelin tissue in diabetic neuropathy has been shown (25). 

The anti-inflammatory and antioxidant effects of *Melilotus Officinalis* have been sufficiently demonstrated in numerous studies both on the cell lines and in the animal models (10, 24 and 26-28). *Melilotus officinalis* extract reduced pro-inflammatory cytokines and oxidative stress in the brain of ischemic rats (24). It also has therapeutic benefits for the treatment of lung injury by inhibiting NF-κB and TLR-4 activities (27). Our results revealed that the immunomodulatory effect of *Melilotus Officinalis* significantly alleviated the expression of pro-inflammatory cytokines (TNF-α, IL-6, IFN-γ, and IL-17). Moreover, it proportionately elevated the expression level of anti-inflammatory cytokines (IL-5). The antioxidant effects of the herbal extract significantly increased the expression level of GPX-1 and CAT in the treatment group. The evaluation of the enzymatic activity of GPX-1 also confirmed the retrieved function of the enzyme in the treatment group compared to the EAE one. The antioxidant potential of *Melilotus Officinalis* was previously reported in iron overload, diabetic foot ulcer, ischemia, aging, and lung injury (11, 12, 17, 24, 27, and 29). Therefore, the dual function of *Melilotus Officinalis* extract could attenuate disease complications in EAE mice by reducing inflammation and oxidative stress.

**Table 1:** Real time PCR primer sequences

**Gene**	**Sequence (5'→3' )**
HPRT-1	F: AGCCCCAAAATGGTTAAGGT R: CAACGGCATATCCAACAACA
TGF-β	F: CAACAATTCCTGGCGTTACC R: GCTGAATCGAAAGCCCTGT
IL-4	F: GTCACAGGAGAAGGGACGC R: AAGCACCTTGGAAGCCCTAC
IL-5	F: CCGCCAAAAAGAGAAGTGTG R: TTGCCCACTCTGTACTCATCA
IL-6	F: ATGATGGATGCTACCAAACTGG R: TATCTCTCTGAAGGACTCTGGC
IL-12	F: TGTCGCTAACTCCCTGCATC R: CTGAGGACACATCCCACTCC
TNF-α	F: GCCCACGTCGTAGCAAACC R: GTCTTTGAGATCCATGCCGTTG
IFN-γ	F: TGAGTATTGCCAAGTTTGAGGTC R: CTGGATTCCGGCAACAGCT
IL-17	F: GCCCTCAGACTACCTCAACC R: TTTCCCTCCGCATTGACAC
CAT	F: CAGCCCTGACAAAATGCTTC R: GCCATCACGCTGGTAGTTG
GPX-1	F:ATCAGTTCGGACACCAGGAG R: TCACTTCGCACTTCTCAAACA

## Conclusion

Overall, our results demonstrated the beneficial effects of *Melilotus Officinalis* herbal extract on healing EAE, through its immunomodulatory and antioxidant properties. This study confirmed the therapeutic potential of *Melilotus Officinalis* in MS and other neurodegenerative diseases.
